# Steroidal Compounds in Commercial Parenteral Lipid Emulsions

**DOI:** 10.3390/nu4080904

**Published:** 2012-08-13

**Authors:** Zhidong Xu, Kevin A. Harvey, Thomas Pavlina, Guy Dutot, Mary Hise, Gary P. Zaloga, Rafat A. Siddiqui

**Affiliations:** 1 Methodist Research Institute, Indiana University Health, Indianapolis, IN 46202, USA; Email: zxu@iuhealth.org (Z.X.); kharvey@iuhealth.org (K.A.H.); 2 Baxter Healthcare Corporation, Deerfield, IL 60015, USA; Email: tom_pavlina@baxter.com (T.P.); mary_hise@baxter.com (M.H.); gary_zaloga@baxter.com (G.P.Z.); 3 Baxter SAS, Maurepas-Cedex 78311, France; Email: dutot.guy@gmail.com; 4 Department of Medicine, Indiana University School of Medicine, Indianapolis, IN 46202, USA

**Keywords:** lipid emulsions, gas chromatography, cholesterol, squalene, phytosterols, sterols

## Abstract

Parenteral nutrition lipid emulsions made from various plant oils contain steroidal compounds, called phytosterols. During parenteral administration of lipid emulsions, phytosterols can reach levels in the blood that are many fold higher than during enteral administration. The elevated phytosterol levels have been associated with the development of liver dysfunction and the rare development of liver failure. There is limited information available in the literature related to phytosterol concentrations in lipid emulsions. The objective of the current study was to validate an assay for steroidal compounds found in lipid emulsions and to compare their concentrations in the most commonly used parenteral nutrition lipid emulsions: Liposyn^®^ II, Liposyn^®^ III, Lipofundin^®^ MCT, Lipofundin^®^ N, Structolipid^®^, Intralipid^®^, Ivelip^®^ and ClinOleic^®^. Our data demonstrates that concentrations of the various steroidal compounds varied greatly between the eight lipid emulsions, with the olive oil-based lipid emulsion containing the lowest levels of phytosterols and cholesterol, and the highest concentration of squalene. The clinical impression of greater incidences of liver dysfunction with soybean versus MCT/LCT and olive/soy lipid emulsions may be reflective of the levels of phytosterols in these emulsions. This information may help guide future studies and clinical care of patients with lipid emulsion-associated liver dysfunction.

## 1. Introduction

Lipid emulsions used for parenteral administration to humans during nutritional support are water-based emulsions made primarily from soybean oil, coconut or palm oil, olive oil and egg yolk phospholipids. These lipid emulsions serve as an important source of energy and essential fatty acids. Soybean oil provides polyunsaturated fatty acids that are rich in the essential fatty acid linoleic acid. Soybean oil-based lipid emulsions are the most common lipid emulsions used in the world today, and the only approved parenteral lipid emulsion in the United States. One of the major complications encountered in patients that are dependent upon parenteral nutrition for survival is the development of parenteral nutrition-associated liver dysfunction (PNALD) [1,2,3,4,5]. PNALD may progress to cirrhosis and liver failure. PNALD has been associated with the use of greater than 1 g/kg/day of soybean oil-based lipid emulsion as part of the parenteral nutrition prescription. The exact etiology of PNALD is unclear, but likely results from multifactorial etiologies that include high intake of lipids, high doses of linoleic acid (found in soybean oil), high intakes of phytosterols, extreme short gastrointestinal tracts, lack of use of enteral nutrition, disruption of the enterohepatic circulation, and recurrent sepsis [1,2,3,4,5,6]. In an attempt to lower the use of soybean oil, lipid emulsions were developed in which soybean oil was replaced with coconut/palm oil (a source of medium-chain fatty acids). A number of commercial preparations of these medium-chain and long-chain triglycerides are available today. Most contain 50% long-chain triglycerides as soybean oil, and 50% medium-chain triglycerides (*i.e.*, MCT/LCT). An alternative lipid preparation is based upon use of olive oil (80%) with soybean oil (20%). Olive oil is rich in the monounsaturated fatty acid oleic acid and low in the omega-6 long-chain polyunsaturated fatty acid linoleic acid. Although poorly studied in clinical trials, there is the clinical perception that both MCT/LCT and olive oil-based lipid emulsions are associated with reduced liver dysfunction [7]. The lowered incidence of liver dysfunction could result from decreased concentrations of linoleic acid, decreased concentrations of polyunsaturated fatty acids, the specific fatty acid composition of the emulsion (*i.e.*, high oleic acid, high medium-chain fatty acids), or decreased levels of phytosterols. 

In addition to triglycerides, these lipid emulsions also contained steroidal compounds. Cholesterol, one of the most important sterols in animals, plays an important role in the regulation of membrane structure and function, and is essential for proper functioning of cells [8,9,10]. Cholesterol is also the major precursor for the production of other molecules, including bile acids and steroid hormones (such as glucocorticoids, mineralocorticoids, estrogens, progesterone, and vitamin D) [8,11]. Moreover, cholesterol has been of great medical importance in recent years because high levels of cholesterol found in lipoproteins have been associated with coronary heart diseases [12,13]. Cholesterol is well absorbed through the gastrointestinal tract, but can also be synthesized by animal cells. 

**Figure 1:**
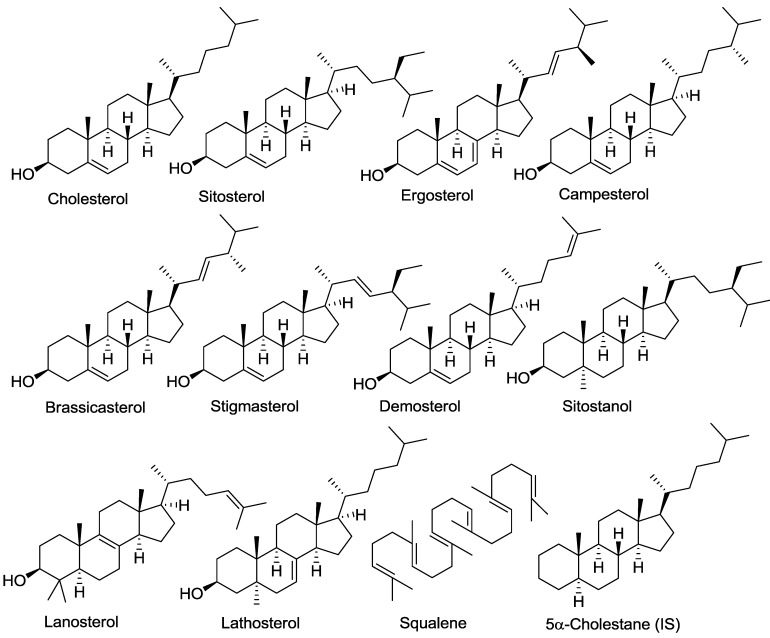
Structure of sterols.

In contrast to cholesterol, plants synthesize sterols (known as phytosterols) that differ slightly from cholesterol through modification of the double bond at C5–C6 and/or the side chain (See Figure 1) [11]. These phytosterols occur naturally in plants and fungi, but are also present in low concentrations in the blood and organs of animals, as a result of plant-based food consumption. Phytosterols are poorly absorbed through the gastrointestinal tract of humans. In human, the ABCG5/8 transporters are responsible in part for the lower absorption rates of plant sterols than that of cholesterol [14]; therefore, the intestine provides an effective barrier against absorption of plant sterols [15,16,17]. These transporters are also expressed in the liver and, also regulate biliary output of both cholesterol and plant sterols [15,16,17]. Phytosterols have also been shown to block cholesterol absorption in the intestines, thus helping to reduce cholesterol levels in humans [18,19,20]. As a result of their cholesterol lowering effects, many enteral products used in the management of patients with cardiovascular diseases contain phytosterols. However, phytosterols and phytostanols are associated with liver toxicity if present in excessive amounts in the circulation [21,22,23]. Thus, knowledge of the phytosterol content of parenteral lipid emulsions can help reduce levels of these compounds in human tissues and may assist in reducing the liver toxicity associated with lipid emulsions. 

Several studies have reported the sterol composition contained in crude oils [24,25]; however, the sterol composition of commercial lipid emulsions, which often employs refined oils, is not widely known. The common procedure used for sterol analysis in food, oil, and biological samples consists of a preliminary extraction of the total lipids with an organic solvent, followed by saponification and isolation of the unsaponifiable fraction, which is then analyzed using a variety of procedures including enzymatic [26,27], colorimetric [28], capillary gas liquid chromatographic (GC) [29,30,31,32], and high performance liquid chromatographic (HPLC) techniques [33,34,35]. Among these analytical techniques, GC and HPLC are more reliable, selective, and accurate because interference from other components can be easily resolved [36,37,38]. Considering the greater resolution offered by GC separation compared to that of HPLC separation, we chose the GC method to assay the sterol content in commercial parenteral lipid emulsions. 

Little is known regarding the sterol composition of commercial lipid emulsions. There are few reports of sterol concentrations of the emulsions in the literature and the manufacturers do not report levels of these compounds in product literature. When levels are reported in the literature, the reports are restricted to one or two emulsions. We could find only one report in the medical literature that compared sterol levels in multiple lipid emulsions. Forchielli *et al.* reported the sterol composition of 7 intravenous lipid emulsions [39]. Unfortunately, these investigators did not report on squalene levels and only measured the levels of three phytosterols (*i.e.*, campesterol, stigmasterol, and β-sitosterol ). In addition, the methodology lacked sufficient details regarding analytical sensitivity and recovery. 

Since phytosterols are implicated in the etiology of PNALD, knowing the levels of phytosterols in various commercial lipid emulsions is important. The objective of the following study was to validate an assay method for determining the concentrations of sterols (cholesterol and phytosterols) and squalene (a sterol precursor) in parenteral lipid emulsions. We chose to determine levels of cholesterol, squalene, and nine different phytosterols in eight different commercial lipid emulsions that represent the most commonly used lipid emulsion types in the world (*i.e.*, soybean oil-predominant, MCT/LCT, and olive oil-predominant emulsions). We also wished to confirm the results of Forchielli *et al*. [39].

## 2. Experimental Section

### 2.1. Chemicals and Reagents

Cholesterol (99.0%) was purchased from Nu-Chek Prep, Inc., USA. β-sitostanol (95.0%) was purchased from Fisher, USA; squalene (98.0%), desmosterol (88.7%), brassicasterol (95.0%), lanosterol (95.0%), ergosterol (95.0%), campesterol (97.0%), stigmasterol (95.0%), β-sitosterol (98.0%), lathosterol (97.0%) and 5α-cholestane (98.0%, internal standard) were purchased from Sigma-Aldrich (St. Louis, MO, USA); and hexane (CHROMASOLV^®^, for HPLC, ≥97.0%), ethanol (anhydrous, 99.8%), and 1,2,3-trihydroxybenzene (pyrogallol, ≥98.0%) were purchased from Sigma-Aldrich (St Louis, MO, USA). A FocusLiner for the GC-2010 system and SACTM-5 (30 m × 0.25 mm × 0.25 μm, Supelco, USA) capillary GC column were purchased from Supelco (Sigma-Aldrich). Lipid emulsions Liposyn^®^ II (50% soybean oil and 50% safflower oil) and Liposyn^®^ III (soybean oil) were from Hospira, Inc. (USA); Lipofundin^®^ MCT (50% soybean oil and 50% MCT) and Lipofundin^®^ N (soybean oil) were from B. Braun (Germany); Structolipid^®^ (64% soybean oil and 36% MCT) and Intralipid^®^ (soybean oil) were from Fresenius Kabi (Germany); and ClinOleic^®^ (80% olive oil and 20% soybean oil) and Ivelip^®^ (soybean oil) were from Baxter Healthcare Corporation (Belgium). The reference and internal standards were dissolved in the hexane solution. All standard stocks were flushed with N_2_ and stored at −20 °C. A 2N ethanolic potassium hydroxide solution (3% pyrogallol) was freshly prepared by dissolving the potassium hydroxide and pyrogallol in 80% ethanol (containing 20% distilled water).

### 2.2. Instrumentation

**Scheme 1:**
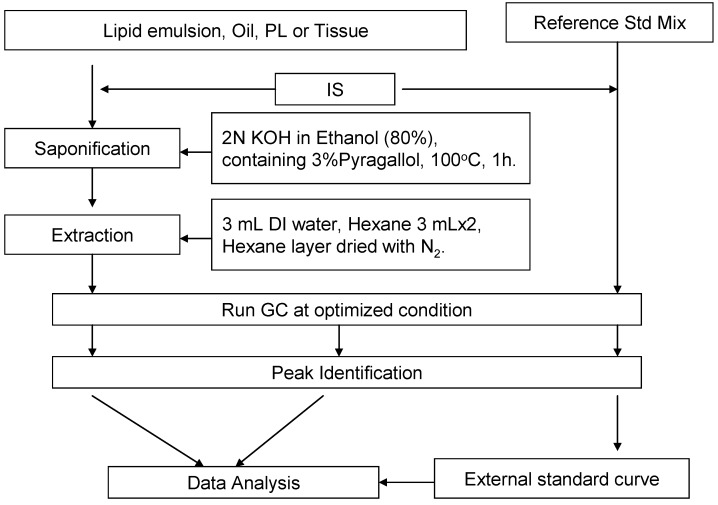
Protocol for sterol analysis.

Based on the known sterol composition existent in vegetable oils, as well as in the egg phosphatide material, we selected 10 sterols and squalene for analysis. The sterols analyzed were cholesterol, β-sitostanol, desmosterol, brassicasterol, lanosterol, ergosterol, campesterol, stigmasterol, β-sitosterol, and lathosterol. The quantification of the individual sterols was preceded by GC analysis using an external standard and internal standard (5α-cholestane). The structures of the tested sterols are shown in Figure 1, and the analytical protocol is illustrated in Scheme 1. 

GC was performed on a Shimadzu GC2010 chromatography system (Shimadzu Scientific Instruments, Columbia, MA, USA) equipped with an auto sampler and a flame ionization detector (FID). A SAC™-5 capillary column (30 m × 0.25 mm × 0.25 μm, Supelco, USA) was used. The injection volume was 1 µL at a split ratio of 1:50 with a split/splitless FocusLiner™. The injection port and detector temperatures were 300 °C. Helium was used as carrier and make-up gases. The column temperature program was as follows: temperature was held at 270 °C for 1 min, increased to 280 °C at 2 °C/min, held at 280 °C for 5 min, increased to 285 °C at 1 °C/min, and finally held at 285 °C for 20 min. 

### 2.3. Saponification and Extraction

The sample preparation was adapted and modified (without derivatization) using previously published methods [37,38,39,40]. Lipid emulsion (200 µL) was added into 10 × 130 mm Pyrex tubes (10 mL) containing Teflon-lined screw caps, and then 20 µL of 5α-cholestane (IS, 0.985mg/mL) and 5 mL of a 2.0 N ethanolic potassium hydroxide solution (3% pyrogallol) were added. The tubes were purged with N_2_ and then tightly closed. The tubes were vortexed gently, subjected to saponification at 100 °C for 1 h, and vortexed twice during the saponification. The tubes were allowed to cool in an ice bath, and then 3 mL of distilled water and 3 mL of hexane were added. The tubes were strongly vortexed and then centrifuged at 900× *g* for 10 min (at 25 °C). The top layer was collected. Another 3 mL hexane was added to the tubes, and the extraction was repeated as described above. The pooled hexane layer was dried under N_2_ flow. Hexane (200 µL) was added to the residues and transferred to a sample vial for GC analysis.

We performed a sample recovery experiment to validate the optimization of extraction and saponification of sterols in the biological samples. Recoveries of the individual sterols after the extraction and saponification were evaluated by spiking reference standard mixtures (containing 50 µg/mL each of squalene, cholesterol, desmosterol, brassicasterol, lathosterol, ergosterol, campesterol, stigmasterol, and β-sitostanol; 300 µg/mL of β-sitosterol; and 100 µg/mL of internal standard) into olive oil, soybean lipid emulsion (Intralipid^®^), and a mouse liver homogenate. The extraction and saponification were carried out as described above. The recoveries were calculated based on the peak area of each individual sterol after normalization with the internal standard. 

### 2.4. External Standard Curve and Equations

We formulated our reference standard mixture based on the sterol composition of the vegetable oil, egg yolk, and the results from our preliminary sterol analysis of the lipid emulsions. Our standard mixture contained 50 µg/mL of brassicasterol; 100 µg/mL each of squalene, desmosterol, lanosterol, ergosterol, campesterol, and lathosterol; 200 µg/mL each of stigmasterol and β-sitostanol; and 500 µg/mL of cholesterol and β-sitosterol. The standard mixture was initially prepared by dissolving the individual reference standards in hexane and then further diluting with hexane (1×, 1/2×, 1/4×, 1/8×, 1/16×, 1/32×, 1/64×, 1/128×, 1/256×, 1/512× and 1/1024×). Analysis was performed by mixing 200 µL of each standard mixture dilution with 20 µL of internal standard (concn. = 0.985 mg/mL). GC analysis was performed using conditions listed above. The peaks were identified by comparisons made using the individual reference standards. The standard curves were generated based on the individual sterol concentration *C* (µg/mL) *vs.* peak area ratio *R* (*R* = A_(Sterol)_/A_(IS)_), using the concentration within the linear range, as listed in Table 1. 

**Table 1:** Method sensitivity (the limit of detection).

Sterols	LOD (µg/mL)	Linear Range (µg/mL)
Squalene	0.2	0.2–100
Cholesterol	0.4	0.4–500
Desmosterol	0.7	0.7–100
Brassicasterol	0.4	0.4–50
Lathosterol	0.7	0.7–100
Ergosterol	0.7	0.7–100
Campesterol	0.4	0.4–100
Stigmasterol	0.7	0.7–200
β-Sitosterol	0.9	0.9–500
β-Sitostanol	0.4	0.4–200
Lanosterol	0.2	0.2–100

Note: GC samples were analyzed using a 1 μL injection, and a split ratio of 50:1.

## 3. Results

### 3.1. Recovery of the Extraction and Saponification

**Figure 2:**
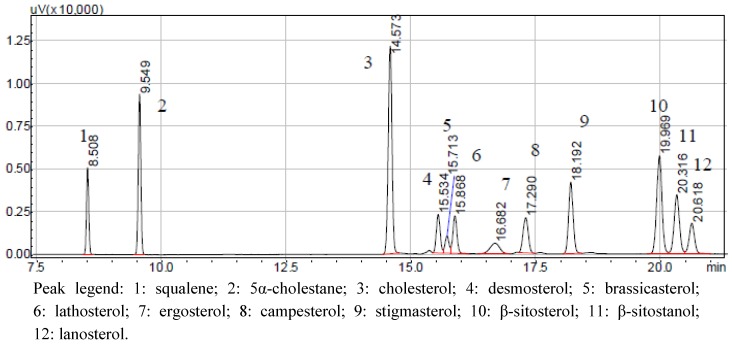
LC chromatograph of the sterol standard mixture.

Using the optimized GC conditions described in the methods section, good resolution of all ten sterols, squalene, and the internal standard were obtained. Furthermore, no interference in resolution of peaks was attributed to the other components contained within the lipid emulsions. A GC chromatograph of the reference standard (Figure 2) clearly shows that lack of derivatization of sterol samples has no effect on peak symmetry, resolution, or quantification. 

The data on sterol recovery analysis is shown in Table 2, which indicates that the saponification and extraction protocol that was used during present studies resulted in at least 95% recovery for most of the sterols. The lowest recovery, 93.2 ± 2.7%, was observed for β-sitostanol in olive oil. Our modified saponification and extraction protocol is fast, easy to handle, and yields accurate results. Our results validated the use of this protocol for sterol determination in plant and animal oils, lipid emulsion, and tissue samples.

**Table 2:** Recovery results of the sterols in samples (%) *.

Sterol	Std. Alone	Std. Spiked in Olive Oil	Std. Spiked in Liver Homogenate	Std. Spiked in Intralipid^®^
IS	100.0 ± 4.6	100.0 ± 4.4	100.0 ± 3.3	100.0 ± 2.8
Squalene	96.8 ± 2.1	95.6 ± 2.6	98.5 ± 1.9	101.3 ± 2.2
Cholesterol	102.7 ± 1.0	94.8 ± 4.0	99.9 ± 5.5	103.2 ± 1.4
Desmosterol	101.6 ± 2.4	98.3 ± 2.5	102.1 ± 2.9	98.3 ± 1.7
Brassicasterol	103.0 ± 1.6	94.9 ± 1.3	96.5 ± 0.7	103.1 ± 1.3
Lathosterol	97.5 ± 3.0	96.5 ± 2.4	99.2 ± 2.8	97.9 ± 2.6
Ergosterol	99.9 ± 0.9	94.1 ± 2.0	100.1 ± 0.5	100.0 ± 1.9
Campesterol	102.3 ± 1.2	94.0 ± 3.0	99.0 ± 8.6	101.7 ± 1.9
Stigmasterol	106.4 ± 1.1	99.5 ± 2.1	101.1 ± 1.0	100.7 ± 1.5
β-Sitosterol	103.1 ± 1.3	97.4 ± 3.7	97.1 ± 1.0	99.8 ± 1.4
β-Sitostanol	94.3 ± 2.8	93.22 ± 2.7	98.5 ± 1.3	95.6 ± 0.7
Lanosterol	98.2 ± 0.9	96.6 ± 2.8	100.5 ± 1.2	97.7 ± 1.7

Abbreviation: Std., standard; * all experiments were conducted in triplicate, and data is shown as mean ± SD.

### 3.2. Sensitivity of the Method

Under the implemented GC conditions, the lowest detection limit (sensitivity) of the method was estimated by determining a concentration of the individual reference standard solution that generated a peak five-fold higher than the baseline noise level. The data is presented in Table 1. The detection limits of various sterols, using this protocol, range from 0.2 to 0.9 µg/mL. The sensitivity for detection of different sterols has the following order: squalene, lanosterol (0.2 µg/mL) > cholesterol, brassicasterol, campesterol, β-sitostanol (0.4 µg/mL) > desmosterol, lathosterol, ergosterol, stigmasterol (0.7 µg/mL) > β-sitosterol (0.9 µg/mL).

### 3.3. Sterol Composition of Lipid Emulsions

**Figure 3:**
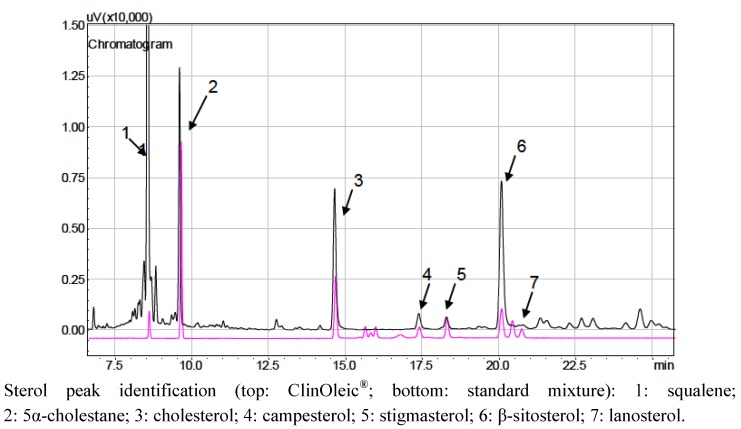
Gas chromatograph of the ClinOleic^®^ lipid emulsion.

The content specifications of the different commercial lipid emulsions used in this study are listed in Table 3. The different sterol components of the lipid emulsions were identified by comparison with reference standards. A representative example using the olive-soybean lipid emulsion (ClinOleic^®^) is presented in Figure 3. The sterol composition of the lipid emulsions is listed in Table 4. The predominant sterols in all of the parenteral lipid emulsions tested were β-sitosterol and cholesterol. The medium-chain triglyceride oil (MCT oil)-containing lipid emulsions (Lipofundin^®^ MCT and Structolipid^®^) contained higher cholesterol levels (219.4 to 290.07 μg/mL) compared to that of the long chain triglyceride plant oil-based lipid emulsions Liposyn^®^ II, Liposyn^®^ III, ClinOleic^®^, and Ivelip^®^ (63.5 to 109.7 μg/mL). Unexpectedly, we found that Lipofundin^®^ N and Intralipid^®^, both containing 100% soybean oil, also contained 217.46 μg/mL and 274.08 μg/mL of cholesterol, respectively. The higher levels of cholesterol in these soybean based lipid emulsions, compared to Liposyn^®^ III and Ivelip^®^, may be due to the different purification processes used for egg phospholipids, with some containing higher concentrations of cholesterol than others. 

**Table 3:** The content specifications of the parenteral lipid emulsions.

Emulsions	Manufacturer	Lot No.	Major component
**Soybean oil-based**			
Intralipid^®^	Fresenius Kabi (DE)	10BK7082	Soybean oil 20 g/100 mL and 1.2 g
Ivelip^®^	Baxter Healthcare Corporation (BE)	08K25A92	Soybean oil 20 g/100 mL and 1.2 g of egg yolk phospholipids
Lipofundin^®^ N	B. Braun (DE)	9173A184	Soybean oil 20 g/100 mL and 1.2 g of egg yolk phospholipids
Liposyn^®^ III	Hospira, Inc. (US)	70913DW	Soybean oil 20 g/100mL and 1.2 g of egg yolk phospholipids
Liposyn^®^ II	Hospira, Inc. (US)	74906DW	Mixture of soybean oil (50%) and safflower oil (50%) 20 g/100 mL, and 1.2 g of egg yolk phospholipids
**Medium- & Long-chain fatty acid-based**			
Lipofundin^®^ MCT	B. Braun (DE)	8494A181	Mixture of soybean oil (50%) and medium-chain structured triglycerides (50%), 20 g/100mL, and 1.2 g of egg yolk phospholipids
Structolipid^®^	Fresenius Kabi (DE)	10CD2533	Interesterified mixture of equimolar amounts of long chain triglycerides and medium chain triglycerides, corresponding to 64% (w/w) and 36% (w/w), respectively.
**Olive oil-based**			
ClinOleic^®^	Baxter Healthcare Corporation (FR)	09D09A91	Mixture of olive oil (approximately 80%) and soybean oil (approximately 20%), 20 g/100 mL, and 1.2 g of egg yolk phospholipids

Abbreviations: BE, Belgium; FR, France; DE, Germany; US, United States.

**Table 4:** Sterol composition of parenteral lipid emulsions (µg/mL ± SD) *.

	Soybean Oil-Based					Medium- & Long-Chain Fatty Acid-Based		Olive Oil-Based
**Sterols**	**Intralipid^®^**	**Ivelip^®^**	**Lipofundin^®^ N**	**Liposyn^®^ III**	**Liposyn^®^ II**	**Lipofundin^®^ MCT**	**Structolipid^®^**	**ClinOleic^®^**
Squalene	7.43 ± 0.10	15.87 ± 0.26	9.18 ± 0.26	8.91 ± 0.41	6.17 ± 0.10	6.08 ± 0.12	4.52 ± 0.02	387.45 ± 2.30
Cholesterol	274.08 ± 3.55	74.92 ± 1.33	217.46 ± 2.40	63.57 ± 2.85	88.99 ± 0.49	219.39 ± 2.93	280.43 ± 0.51	109.70 ± 0.38
Desmosterol	ND	ND	ND	ND	ND	ND	ND	ND
Brassicasterol	ND	ND	ND	ND	ND	ND	ND	ND
Lathosterol	ND	ND	ND	ND	ND	ND	ND	ND
Ergosterol	ND	ND	ND	ND	ND	ND	ND	ND
Campesterol	55.35 ± 0.48	51.59 ± 1.19	85.85 ± 0.97	94.26 ± 4.30	68.12 ± 0.60	30.87 ± 0.62	43.96 ± 0.39	13.33 ± 0.12
Stigmasterol	65.05 ± 0.52	59.41 ± 1.25	96.50 ± 0.88	93.41 ± 3.67	60.93 ± 0.65	46.00 ± 0.95	48.80 ± 0.31	12.15 ± 0.04
β-Sitosterol	302.64 ± 2.00	277.04 ± 5.61	420.32 ± 4.73	390.65 ± 18.68	339.78 ± 2.62	191.63 ± 3.18	240.04 ± 0.61	240.59 ± 2.08
β-Sitostanol	7.68 ± 0.25	6.88 ± 0.15	10.06 ± 0.39	8.27 ± 0.39	10.45 ± 0.30	5.67 ± 0.12	6.77 ± 0.25	4.57 ± 0.16
Lanosterol	8.35 ± 2.48	7.49 ± 0.34	9.13 ± 0.39	11.78 ± 0.62	8.50 ± 0.34	3.96 ± 0.22	6.28 ± 0.08	3.74 ± 0.20
Total sterols	713.15 ± 9.27	477.33 ± 9.87	839.31 ± 9.75	661.93 ± 30.50	576.77 ± 5.00	497.52 ± 8.02	626.28 ± 2.15	384.08 ± 2.98
Total phytosterols	439.07 ± 5.72	402.41 ± 8.55	621.85 ± 7.36	598.37 ± 27.65	487.77 ± 4.51	278.14 ± 5.09	345.85 ± 1.64	274.38 ± 2.60
Phytosterols (mg) per 100 g of lipid	220 ± 2.86	201 ± 4.25	311 ± 3.68	299 ± 13.82	249 ± 2.13	139 ± 2.55	173 ± 0.82	137 ± 1.30

Abbreviation: ND, Not Detected; * all experiments were conducted in triplicate, and data is shown as mean ± SD; lipid emulsions analyzed are representative of only a single Lot number.

The predominant phytosterol in these emulsions was β-sitosterol, which is the major phytosterol in plant oils (*i.e.*, soybean oil, olive oil, sunflower oil, *etc.*). The content of β-sitosterol in the lipid emulsions ranged from 191.63 μg/mL (Lipofundin^®^ MCT) to 420.32 μg/mL (Lipofundin^®^ N). We also found campesterol and stigmasterol in most plant oil-based lipid emulsions to be in a nearly 1:1 ratio. In addition, we were also able to detect lower levels of phytosterols and phytostanols, including lanosterol and β-sitostanol, in the lipid emulsions. However, we did not find detectable amounts of desmosterol, brassicasterol, and ergosterol. Total phytosterol content of the lipid emulsions varied greatly from 274 to 622 μg/mL (Table 4). Concentrations were lowest (<300 μg/mL) in ClinOleic^®^ (274.38 μg/mL) and Lipofundin^®^ MCT (278.14 μg/mL), intermediate (300–400 μg/mL) in Structolipid^®^ (345.85 μg/mL), and highest (>400 μg/mL) in Ivelip^®^ (402.41 μg/mL), Intralipid^®^ (439.07 μg/mL), Liposyn^®^ II (487.77 μg/mL), Liposyn^®^ III (598.37 μg/mL), and Lipofundin^®^ N (621.85 μg/mL). The quantity of phytosterols that a patient would receive per day if given 100 g of lipid per day ranged from 137 mg to 311 mg (Table 4).

We also detected the presence of squalene in the different plant-based oil emulsions. The olive oil-based lipid emulsion, ClinOleic^®^, contained 387.45 μg/mL of squalene. Ivelip^®^ had low amounts of squalene (15.87 ± 0.26), whereas all other lipid emulsions had a squalene content under 10 µg/mL. 

## 4. Discussion

The current study validates the assay used to measure cholesterol, phytosterols, and squalene in a variety of commercially available parenteral lipid emulsions. The assay produced good separation of the various phytosterols measured, had sensitivity for detection of the various phytosterols of 0.2–0.9 μg/mL, and a recovery of 93%–103%. We measured the levels of the various sterols and squalene in eight of the most commonly used lipid emulsions that are based upon soybean oil, coconut/soybean oils, and olive/soybean oils. Concentrations of cholesterol, phytosterols, and squalene varied greatly between the different lipid emulsions. Cholesterol varied from 63.57 to 280.43 μg/mL (441% variation); total phytosterols varied from 384.08 to 839.31 μg/mL (219% variation); and squalene varied from 4.52 to 387.45 μg/mL (8572% variation). β-Sitosterol levels were the highest amongst the phytosterols, followed by stigmasterol and campesterol. The large variations of concentrations of these compounds, all of which possess biologic activity, may have clinical significance upon efficacy and safety of the various lipid emulsions. Future studies should be performed to determine the exact effects of the various sterols and squalene upon biological functions. 

Cholesterol is an important component of cell membranes and lipoproteins. High levels of cholesterol found in low density and very low density lipoproteins are associated with an increased risk of cardiovascular complications. A variety of drugs are available that reduce levels of cholesterol (*i.e.*, statins) and decrease the risk of cardiovascular disease. On the other hand, very low levels of cholesterol occurring in underfed individuals are also associated with increased morbidity and mortality. Thus, the optimal levels of dietary intake of cholesterol for individuals with different diseases remain unclear. 

Levels of cholesterol intake in most patients consuming weight maintaining diets is more than adequate for tissue functions, since cholesterol is found in most foods of animal origin. Median cholesterol intakes in western diets range from 250 to 325 mg/day in men and 180 to 205 mg/day in women [41]. Levels of cholesterol intake with the different lipid emulsions would range from 33 to 140 mg per day in individuals consuming 100 g of lipid (approximately 1000 Kcal). However, many parenteral nutrition-dependent patients receive substantially lower amounts of lipid each day (*i.e.*, 20–60 mg/day), and these patients would receive significantly less cholesterol. Cholesterol is not required in the diet of humans, since tissues can synthesize cholesterol. Thus, there is no recommended “Adequate Intake” or “Recommended Dietary Allowance” for cholesterol [41]. However, the ability of the tissues of patients with severe organ injuries, infection, systemic inflammation and/or starvation to synthesize cholesterol is unclear, and the exact biological consequences of low intakes of cholesterol in various patient groups require further investigation.

Hepatobiliary complications are a major cause of morbidity and mortality in patients receiving long-term parenteral nutrition [2,3,4,5]. The pathogenesis of PNALD is likely multifactorial. Proposed mechanisms include alterations in cholesterol metabolism, bile composition and/or bile transport, lack of enteral gastrointestinal stimulation due to short bowel (with loss of trophic factors and other gut derived hormones; loss of the enterohepatic circulation of bile acids), endotoxins or exotoxins released into the circulation due to loss of gut barrier functions, recurrent systemic infection, and direct hepatotoxicity from substances such as phytosterols in the lipid emulsions [1,2,3,4,5,42].

The mechanisms by which phytosterols may cause liver disease are unclear. However, phytosterols have been reported to inhibit enzymes involved in cholesterol and bile acid synthesis and metabolism [11,43,44,45]. Inhibition of bile acid synthesis would limit excretion of phytosterols and bilirubin, leading to their accumulation in the liver [46]. Cholestasis and liver injury could be the result. Infants may be particularly susceptible to liver injury by phytosterols due to reduced bile acid synthesis and immature bile secretory systems [4]. Sterols are important constituents of cell membranes and modulate membrane fluidity, cell membrane proteins such as transporters, and endocytosis and exocytosis. Replacement of membrane cholesterol with phytosterols may alter membrane functions [4] that interfere with cell function. For example, after intravenous injection, β-sitosterol and campesterol accumulate in the liver and may replace 20%–60% of hepatic microsomal cholesterol [4]. Phytosterols may also interfere with cholesterol metabolism [4]. For example, β-sitosterol and cholestanol can inhibit cholesterol-7α-hydroxylase, the rate limiting enzyme required for the conversion of cholesterol to bile acids [4,44,45]. Farnesoid X receptor (FXR) is a nuclear receptor that acts as a bile acid sensor that maintains safe intrahepatic bile acid levels [47]. FXR reduces bile acid import through the hepatic sinusoids, decreases bile acid synthesis, and increases bile acid efflux across canalicular and sinusoidal membranes. Mice lacking the receptor are ultrasensitive to bile acid-induced hepatic injury while FXR agonists protect from cholestasis [47]. Stigmasterol, a phytosterol present in plant derived lipid emulsions, is a potent antagonist of FXR [47]. Stigmasterol has also been reported to antagonize the nuclear receptor PXR (pregnane X receptor), which plays a role in detoxifying the liver from excess bile acids [47]. These effects of phytosterols may result in hepatic dysfunction, but they may also interfere with the function of other cells such as erythrocytes and leukocytes.

The presence of elevated levels of phytosterols in the circulation during administration of parenteral lipid emulsions has been associated with the development of liver dysfunction in patients treated with parenteral nutrition [6,21,22,23,48]. Studies by Clayton *et al.* [23], in children on long-term parenteral lipid infusion, reported high levels of plasma phytosterols that were associated with cholestatic liver disease. Clayton *et al.* [23] also associated increased phytosterols levels with the development of thrombocytopenia and reported improvement in liver disease and platelet counts when intake of the lipid emulsions was reduced. Ellegard *et al.* [21] reported a significant elevation in plasma phytosterol levels in adults with short bowel receiving parenteral nutrition with lipid emulsion compared to healthy controls and short bowel patients not requiring parenteral nutrition. However, these authors did not relate the levels to liver dysfunction. More recently, Llop *et al*. [22] also reported the development of liver dysfunction in adult home parenteral nutrition patients infused with parenteral lipid emulsion. Bilirubin and aspartate aminotranferase levels in the blood of the patients significantly correlated with blood phytosterols concentrations, and blood levels in the patients receiving parenteral nutrition were significantly higher than healthy controls on normal diets (55.4 *vs.* 14.8 μg/mL). Hallikainen *et al.* [48] reported on a patient with parenteral nutrition-associated liver disease. When the patient was switched from a soybean oil lipid emulsion to an olive/soybean oil lipid emulsion, the concentrations of phytosterols were lowered while, simultaneously, the liver enzymes returned to lower values. Lowering the intake of soybean based lipid emulsions that contain high levels of phytosterols has also been shown to reduce the incidence of liver disease [1]. Others have reported resolution of liver disease when patients receiving soybean oil lipid emulsions were switched to a lipid emulsion based upon fish oil (fish contains very low levels of phytosterols) [49]. These studies indicate an association between high intakes of parenteral phytosterols and the development of liver dysfunction. Our study indicates that β-sitosterol, stigmasterol, and campesterol are the three phytosterols found at the highest concentrations in the various plant based commercial lipid emulsions. Future studies should evaluate these three phytosterols for their effects upon liver integrity. Clearly, there is a need to perform animal studies that directly link the various phytosterols to liver toxicity. Most data in the literature represent associations of liver disease with lipid emulsion administration, but the exact etiology remains elusive. A linking of phytosterols with liver disease would provide impetus for lipid emulsion manufacturers to remove or reduce levels of the phytosterols in their lipid emulsions.

Western diets provide approximately 150–350 mg/day of phytosterols, while vegetarian diets provide up to 500 mg/day [22]. Approximately 5% of plant sterols are absorbed from the diet by the small intestine [22,48], representing approximately 5–17 mg/day of absorbed phytosterol. The absorption of phytosterols contrasts with that of cholesterol, where approximately 55% is absorbed. Most of the phytosterols are transported into the portal circulation and metabolized by the liver [48]. Phytosterols are normally excreted in the bile. During parenteral administration, phytosterols enter the systemic circulation through the vena cava, bypassing first pass hepatic elimination. Thus, phytosterols given parenterally result in higher levels in non-hepatic tissues than when given via the gastrointestinal tract. Total phytosterol intake from the lipid emulsions ranged from 137 mg/day (ClinOleic^®^; olive/soy lipid emulsion) to 311 mg/day (Lipofundin^®^ N; soybean lipid emulsion) in patients receiving 100 g of lipid per day (Table 4). These intakes are substantially higher than the typical daily amounts that enter the circulation from the diet, and differ greatly depending upon the plant source used to manufacture the lipid emulsion. The intakes remain high even with more moderate intake of lipid (*i.e.*, 50 g/day). Since the duration and dose of lipid emulsion administration are associated with the development of liver dysfunction [1,2], the choice of lipid emulsion (*i.e.*, using an emulsion with lower levels of phytosterols) may have clinical significance in limiting the development of liver injury. Decreasing intake of phytosterols by lowering the dose of lipid emulsion administered has been shown to limit and reverse the degree of hepatic dysfunction [1]. In addition, the clinical impression of greater incidences of liver dysfunction with soybean versus MCT/LCT and olive/soy lipid emulsions may be reflective of the levels of phytosterols in these emulsions. These observations support but do not prove that phytosterols in the lipid emulsions are the cause of the liver disease. Future studies should compare the incidence of liver dysfunction using lipid emulsions with high versus low levels of phytosterols. 

Our results differ slightly from the results of Forchielli *et al.* [39], who measured the levels of cholesterol, β-sitosterol, campesterol, and stigmasterol in seven commercial lipid emulsions. Our results confirm that β-sitosterol, campesterol, and stigmasterol are the most common sterols in commercial lipid emulsions. However, we also detected significant quantities of β-sitostanol and lanosterol in the lipid emulsions. Our results also indicate that the lipid emulsions lack detectable quantities of desmosterol, brassicasterol, lathosterol, and ergosterol. We detected squalene in all of the lipid emulsions; levels were significantly higher in the olive oil emulsion compared to the soy and soy/coconut emulsions. In general, we detected slightly higher levels of phytosterols in the emulsions (approximately 13%–40%) than those reported by Forchielli *et al.* [39]. Some of this variability relates to levels of the two additional phytosterol compounds that we measured. Other variability may result from use of different batches and assays. Overall, the relative differences in sterol concentrations between emulsions are similar in our study and the study of Forchielli *et al.* [39].

Squalene is an open-chain, 30-carbon isoprenoid that is produced in both plants and animals [50]. Squalene can be cyclized in different ways and can serve as a precursor for the synthesis of 20 different compounds, including cholesterol, steroid hormones, and vitamin D in the human body, and dammarane, hopane, oleanane and friedelane in plants and microorganisms [51]. Interestingly, studies in rabbits and humans found that high intakes of squalene are not associated with an increased risk of atherosclerosis, despite the fact that dietary intake of squalene can augment cholesterol and bile acid production [52]. On the contrary, studies have shown that long-term increases in squalene from rat and human diets fails to elevate serum cholesterol [53,54]. Furthermore, squalene inhibits activity of β-hydroxy-β-methylglutaryl-CoA reductase (HMGCoA reducatse), a regulatory enzyme in cholesterol biosysnthesis. Inhibition of this enzyme is felt to have antiatherosclerotic activity [55]. Squalene has been reported to have other functional activities, that include acting as a scavenger for reactive oxygen species [50,56], anti-inflammatory properties [50], anticancer effects (particularly for skin cancers) [52,57,58,59,60], and coadjuvant effects when administered with vaccines [51]. Further studies are needed to evaluate potential benefits of squalene in patients receiving parenteral nutrition. 

In this report, we evaluated levels of phytosterols in lipid emulsions based upon plant oils. However, some recent lipid emulsions also include fish oils. Fish oils do not contain significant levels of phytosterols, since they originate from animal sources where the primary sterol is cholesterol. In preliminary analyses, we measured the level of phytosterols and cholesterol in a fish oil emulsion (Omegaven^®^, Fresenius Kabi, Germany). Total sterol levels were comparable between Omegaven^®^ and the olive oil-based lipid emulsion, ClinOleic^®^ (372.9 *vs.* 384.1 mg/L, respectively). However, the distribution of sterols was significantly different between the two emulsions. Omegaven^®^’s content included almost exclusively cholesterol, while ClinOleic^®^ contained a mixture of cholesterol and phytosterols. Omegaven^®^ was also low in squalene (26.7 mg/L). In addition, we analyzed a lipid emulsion based upon a mixture of soybean oil, medium-chain triglycerides, olive oil, and fish oil (SMOFlipid^®^, Fresenius Kabi, Germany). SMOFlipid^®^ contained a total sterol content of 657 mg/L, composed of a mixture of cholesterol (450 mg/L) and phytosterols (207 mg/L). The squalene content of SMOFlipid^®^ was 52 mg/L.

## 5. Conclusion

Our detailed analysis of phytosterols in various parenteral lipid emulsions indicates that Lipofundin^®^ MCT and ClinOleic^®^ have the lowest levels of phytosterols. Among these, ClinOleic^®^, an 80% olive- and 20% soybean-lipid emulsion, is very unique in that it has the lowest level of cholesterol and the highest level of squalene. Since levels of phytosterols differ significantly in various lipid emulsions, further studies are suggested to determine the types and quantities of phytosterols that may cause tissue toxicity following intravenous administration.
